# Analyzing the Anatomical Characteristics of the Facial Artery Branches: A Human Cadaveric Study

**DOI:** 10.7759/cureus.71149

**Published:** 2024-10-09

**Authors:** Getsy Metilda, Lakshmi Rathan A C, Pratheepa S Natarajan, Vivek N, Jyotsna Rajan

**Affiliations:** 1 Department of Oral and Maxillofacial Surgery, Sri Ramaswamy Memorial (SRM) Kattankulathur Dental College and Hospital, Sri Ramaswamy Memorial Institute of Science and Technology (SRMIST), Chengalpattu, IND; 2 Department of Anatomy, Sri Ramaswamy Memorial (SRM) Medical College Hospital and Research Center, Sri Ramaswamy Memorial Institute of Science and Technology (SRMIST), Chengalpattu, IND

**Keywords:** cadaveric study, dissection, facial artery, submental artery, superior labial artery

## Abstract

Objectives: The facial artery is the predominant branch of the external carotid artery supplying blood to the head and neck regions. Knowledge regarding the anatomical path and distribution of each facial artery's branches is essential. The aim of the study is to assess the levels, establish the reference positions of each branch of the facial artery to reliable landmarks in the face and neck regions, and evaluate the outer diameter and linear measurement.

Materials and methodology: A prospective single-center cadaveric study was done on 60 hemifaces from 30 properly embalmed and formalin-fixed male cadavers. The statistical analysis was carried out using IBM SPSS Statistics for Windows, Version 22 (Released 2013; IBM Corp., Armonk, New York, United States). Descriptive statistics and inferential statistics have been applied. Nonparametric analysis and the tests of normality were used. The Wilcoxon signed-rank test was used to compare the various parameters between the left and right sides of each branch of the facial artery.

Results: The average length and diameter of each branch of the facial artery measured in millimeters are as follows: superior labial, 27.3 ± 9.17 mm and 1.6 ± 0.2 mm; inferior labial, 31.5 ± 7.86 mm and 1.3 ± 0.6 mm; lateral nasal, 10.6 ± 4.55 mm and 0.74 ± 0.2 mm; angular, 29.65 ± 7.93 mm and 0.8 ± 0.2 mm; and submental, 35.9 ± 5.3 mm and 0.9 ± 0.4 mm. Statistically, there is no remarkable variation between the left and right sides except the submental artery, which shows a remarkable difference based on the origin between the left side and right side with a p-value of 0.039.

Conclusion: This study's findings will enable surgeons to more efficiently plan and design reconstructive flaps based on the facial artery, pertaining to this specific ethnic population (Indian origin), which is not available in published history until now.

## Introduction

The facial artery is the predominant branch of the external carotid artery, supplying blood to the head and neck regions. The artery is present superficially in the skin, subcutaneous cheek fat, and the muscles [1,2]. The neck is enclosed by the skin, platysma, and fasciae and runs all the way down to the digastric and stylohyoid muscles [3,4]. It then follows down to the border of the mandible and reaches the floor of the mandible; the artery curves around its inferior border, which is present anteriorly to the masseteric muscle, and enters the face [5-7]. 

Some of the facial artery branches in the neck include the tonsillar, ascending palatine, glandular, and submental branches [6,8]. The ascending palatine artery emerges near the facial artery’s point of origin [9-12]. The tonsillar artery behaves as the primary source of blood for the palatine tonsil. The ascending palatine artery can occasionally give birth to the tonsillar artery [13,14]. The submental artery is the facial artery’s largest branch, which is present cervically [15-17]. This provides blood flow to the surrounding skin and muscles [1]. The glandular branches are large vessels that nourish the submandibular salivary gland, surrounding muscles, skin, and lymph nodes [5]. 

The facial artery provides branches to the facial muscles and skin, and some of the branches are named superior labial, lateral nasal, and inferior labial arteries. It merges with the ophthalmic artery, which is a dorsal nasal branch, and turns into the angular artery near the eye’s medial angle [18]. The inferior labial artery emerges immediately beneath the commissure of the lip, and it terminates with an anastomosis on the contrary side of the artery and a branch rising from the inferior alveolar artery [6]. The superior labial artery is said to be the largest and most tortuous when compared with the inferior labial, which mainly supplies the upper part of the lip and the nasal septum that produces a septal and alar branch [7]. The lateral nasal artery is emitted beside the nose, and that would be restored by a branch from the superior labial artery [1]. The angular artery is the portion of the artery that is situated distant to its terminal branch beyond the lateral nasal artery [1].

Due to its superficial course, the facial artery is more likely to be hurt in a variety of severe events, such as ballistic or gunshot wounds, accidents, and tumor ablative operations. Understanding the angio-architecture helps to design and plan pedicled flaps, preserve tissue, and perform better surgery [19]. Anatomical information of the facial artery is also important in radio-imaging to understand and interpret the face angiography [8,20]. 

The facial artery is the most important vascular structure for a maxillofacial surgeon with many procedures relying on the path and pattern, such as reconstructive procedures, cosmetic injections, and preventing massive hemorrhage due to transection of the artery, embolization, etc. Hence, in-depth analysis and knowledge of the same are necessary [21,22].

In the literature, various studies have reported on the emphasis on variation in the genesis, the facial artery branching anatomy, and trajectories [23,24]. However, there exists a knowledge gap regarding the levels at which the facial artery gives off branches and its relationship to reliable landmarks in the head and neck regions. The aim of this study is to assess the levels at which the facial artery gives off its branches and to establish a reference position of each branch to reliable landmarks in the face and neck regions. The secondary objective is to evaluate the outer diameter and linear measurement of each branch of the facial artery. 

## Materials and methods

**Figure 1:**
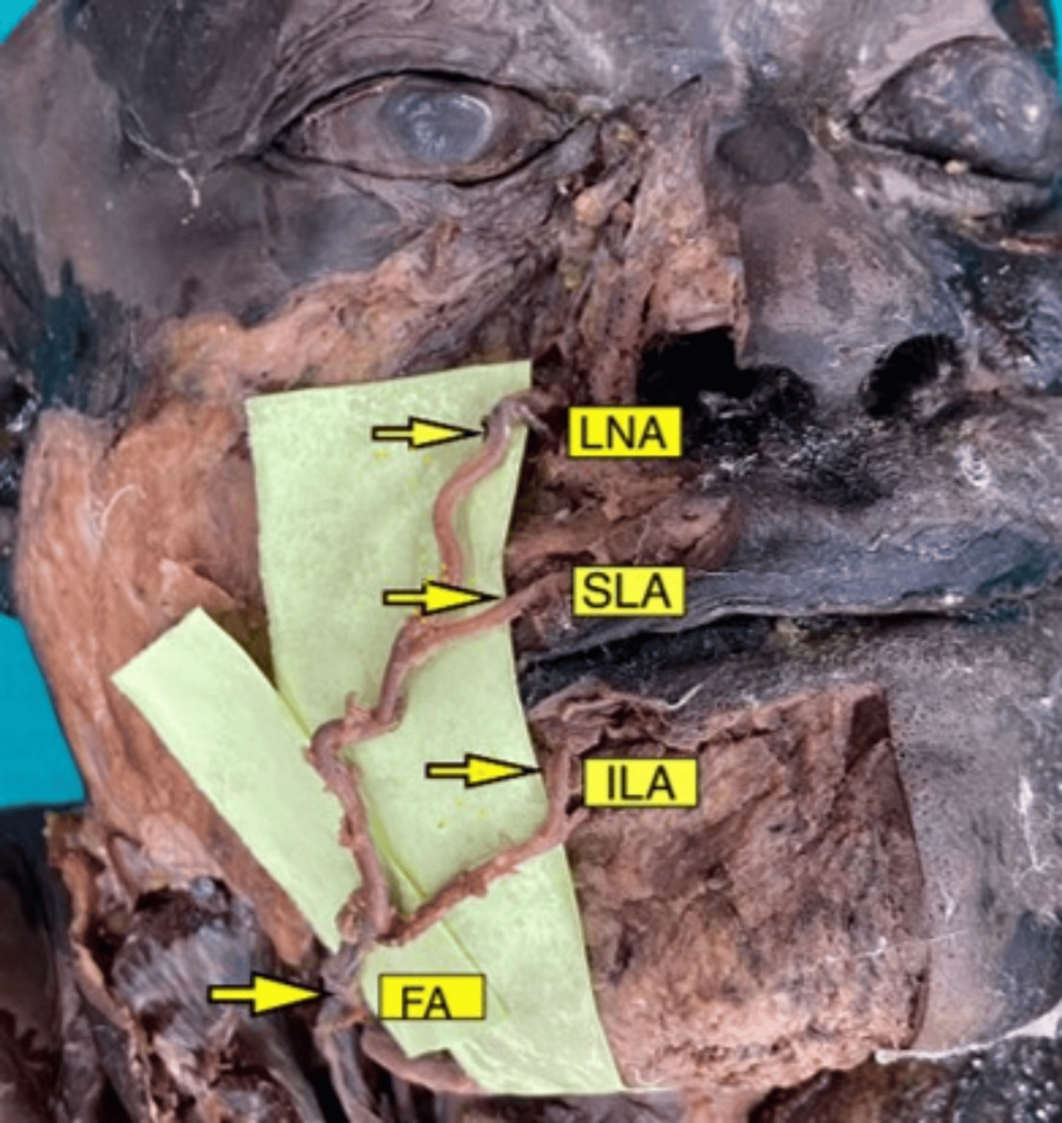
Branches of the facial artery on the right side of the face Image scaled to 1.5x magnification for better visualization of structures. FA: facial artery, ILA: inferior labial artery, SLA: superior labial artery, LNA: lateral nasal artery.

**Figure 2:**
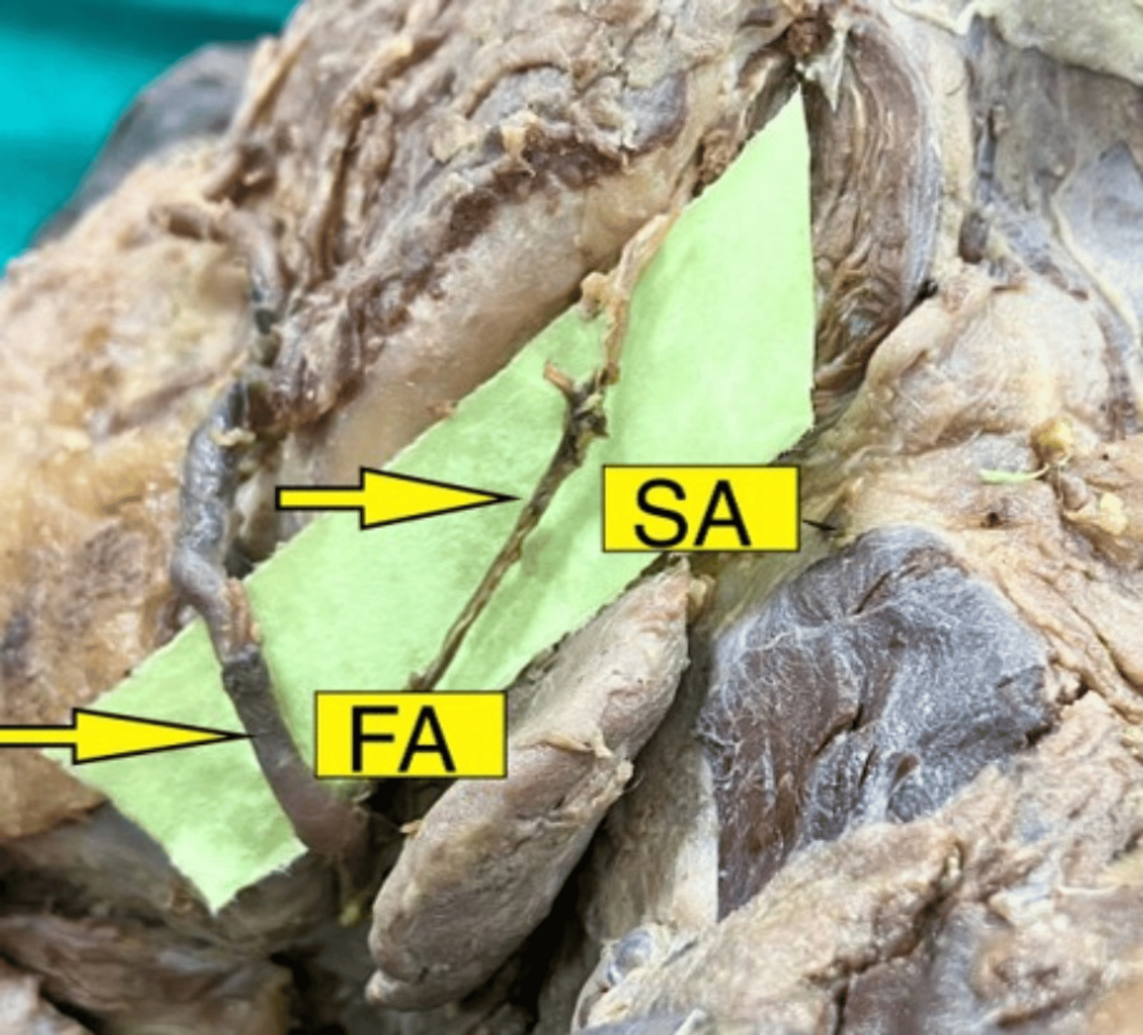
Submental branch of the facial artery in the right side of the neck Image scaled to 1.5x magnification for better visualization of structures. FA: facial artery, SA: submental artery.

A prospective single-center cadaveric study was done on 60 hemifaces from 30 properly embalmed and formalin-fixed cadavers in the Department of Anatomy, Sri Ramaswamy Memorial (SRM) Kattankulathur Medical College Hospital and Research Centre. The study was carried out after approval by the Institutional Ethics Committee [SRMIEC-ST0923-721] under the Indian Council of Medical Research-Short-Term Studentship (ICMR-STS program) (reference ID: 2023-13243) for a period of two months. The age and gender of the cadaver were not considered. The cadaveric hemifaces were grouped into the left side as Group 1 and the right side as Group 2, to compare the variation in the pattern of branching, termination, and linear measurements of the facial artery and its branches. Dissection of all the specimens was carried out by following Cunningham’s Manual of Practical Anatomy Volume 3 (16th Edition) [25]. The dissection was done carefully in such a way that the entire facial artery course from its origin to the termination, along with its branches, is identified and preserved. Mutilated bodies were excluded from the study. A midline incision was made. The skin, subcutaneous tissue, platysma, and fascia present superficially covering the carotid triangle were reflected laterally on both sides of the neck. After removing the cervical fascia, which is present deep, the boundaries of the omohyoid and sternocleidomastoid were established. After determining the boundaries of the digastric posterior belly muscle and the stylohyoid muscle above, the submandibular gland was mobilized. The external carotid artery and its branches have been found after the carotid sheath was reflected in the carotid triangle. The facial artery, the third branch emerging from the external carotid in the neck, was situated deep to the mandible and continued distally in its course [26,27]. Subsequently, the genesis of the facial artery was traced and has been noted (Figure 1 and Figure 2).

In this study, the following criteria have been evaluated: (1) The level of branching of every branch of the facial artery in the face and neck from the reliable landmark was measured. (2) The length of each branch of the facial artery was measured using a vernier caliper. (3) The distance of each branch of the facial artery to the reliable landmarks has been calibrated. The reliable landmarks considered in the face are the commissure of the lip, alar base, menton, and gonion (Table 1). (4) The pattern of origin and termination of the facial artery according to the categorization by Bayram et al. was noted [28].

**Table 1:** Description of reliable landmarks of the face

S. no.	Landmark	Description
1.	Menton	A triangular projection in the external surface of the mandible in the midline
2.	Commissure of the lip	The junction of the vermillion border of the superior labium meets that of the inferior labium
3.	Gonion	A maximum curvature in the posterior mandibular region where the ascending ramus becomes the body of the mandible
4.	Ala of nose	The tissue comprising the lateral boundary of the nose, inferiorly surrounding the nares

The statistical analysis was carried out using IBM SPSS Statistics for Windows, Version 22 (Released 2013; IBM Corp., Armonk, New York, United States). Descriptive statistics and inferential statistics have been applied and do not show a normal distribution; therefore, nonparametric analysis and the tests of normality were used. The Wilcoxon signed-rank test was used to compare the various parameters between the left and right sides of each branch of the facial artery.

## Results

Origin, length, diameter, location to its reliable landmark, and termination of each branch of the facial artery

Superior Labial Artery

The average length of the superior labial artery was 27.3 ± 9.17 mm with a diameter of 1.68 ± 0.42 mm. The level of branching of the superior labial artery out from the commissure of the lip was measured to be 21.03 ± 9.8mm, and the location to its closest landmark was 14.14 ± 5.05 mm (Table 2). Statistically, the superior labial artery does not have any significant difference based on the length, diameter, location to the landmark, or origin between the left and right sides.

**Table 2:** Mean comparison between the left and right sides of the superior labial artery using the Wilcoxon signed-rank test SLA: superior labial artery.

Parameter	Group 1		Group 2		Z value	p-value
	Mean	Std. D.	Mean	Std. D.		
SLA length	29.06	8.04	25.6	10.03	-1.1	0.271
SLA diameter	1.67	0.46	1.7	0.38	-0.75	0.455
SLA location to the landmark commissure of the lip	14.81	5.34	13.41	4.77	-0.96	0.336
SLA origin	19.82	9.29	22.24	10.31	-1.34	0.181

Inferior Labial Artery

The average length of the inferior labial artery was 31.5 ± 7.86 mm with a diameter of 1.33 ± 0.6 mm. The level of branching of the inferior labial artery from the commissure of the lip was measured to be 31.2 ± 6.49 mm, and the location to its closest landmark was 22.62 ± 6.69 mm (Table 3). Statistically, the inferior labial artery does not have any significant difference based on the length, diameter, location to the landmark, or origin between the left and right sides.

**Table 3:** Mean comparison between the left and right sides of the inferior labial artery using the Wilcoxon signed-rank test ILA: inferior labial artery.

Parameter	Group 1		Group 2		Z value	p-value
	Mean	Std. D.	Mean	Std. D.		
ILA length	31.27	6.78	31.86	8.94	-0.288	0.773
ILA diameter	1.41	0.56	1.25	0.65	-0.747	0.455
ILA location to the landmark commissure of the lip	21.19	7.04	24.05	6.53	-1.368	0.171
ILA origin	31.96	6.35	30.56	6.63	-0.607	0.544

Lateral Nasal Artery

The average length of the lateral nasal artery was 10.6 ± 4.5 mm with a diameter of 0.74 ± 0.2 mm. The level of branching of the lateral nasal artery from the commissure of the lip was measured to be 10 ± 6.8 mm, and the location to its closest landmark was 1.15 ± 1.25 mm (Table 4). Statistically, the lateral nasal artery does not have any significant difference based on the length, diameter, location to the landmark, or origin between the left and right sides.

**Table 4:** Mean comparison between the left and right sides of the lateral nasal artery using the Wilcoxon signed-rank test LNA: lateral nasal artery.

Parameter	Group 1		Group 2		Z value	p-value
	Mean	Std. D.	Mean	Std. D.		
LNA length	11.22	4.85	10.04	4.25	-1.142	0.254
LNA diameter	0.72	0.2	0.76	0.2	-0.61	0.542
LNA location to the landmark to the alar base	1.04	1.09	1.26	1.42	-0.77	0.441
LNA origin	9.43	6.76	10.57	6.98	-0.979	0.328

Angular Artery

The average length of the angular artery was 29.6 ± 7.93 mm with a diameter of 0.85 ± 0.27 mm. The level of branching of the angular artery from the commissure of the lip was measured to be 16.6 ± 5.3 mm, and the location to its closest landmark was 7.76 ± 3.08 mm (Table 5). Statistically, the angular artery does not have any significant difference based on the length, diameter, location to the landmark, or origin between the left and right sides.

**Table 5:** Mean comparison between the left and right sides of the angular artery using the Wilcoxon signed-rank test AA: angular artery.

Parameter	Group 1		Group 2		Z value	p-value
	Mean	Std. D.	Mean	Std. D.		
AA length	29.98	8.01	29.32	7.85	-0.283	0.777
AA diameter	0.84	0.29	0.87	0.25	-0.719	0.472
AA location to the landmark lateral to the alar base	7.56	2.94	7.96	3.22	-0.458	0.647
AA origin	15.34	7.47	17.99	9.21	-1.211	0.226

Submental Artery

The average length of the submental artery was 35.9 ± 5.3 mm with a diameter of 0.94 ± 0.44 mm. The level of branching of the submental artery from the commissure of the lip was measured to be 17.56 ± 4.68 mm, and the location to its closest landmark was 19.35 ± 5.38 mm (Table 6). Statistically, the submental artery does not have any significant difference based on the length, diameter, location to the landmark, or origin between the left and right sides but shows significant difference based on the origin between left and right sides with a p-value of 0.039.

**Table 6:** Mean comparison between the left and right sides of the submental artery using the Wilcoxon signed-rank test SA: submental artery. *Statistically significant difference present in the origin of the submental artery between the left and right sides (p-value, 0.039).

Parameter	Group 1		Group 2		Z value	p-value
	Mean	Std. D.	Mean	Std. D.		
SA length	36.12	6.17	35.87	4.43	-0.206	0.837
SA diameter	0.93	0.45	0.96	0.43	-0.18	0.855
SA location to the landmark gonion	18.63	5.28	20.07	5.49	-1.05	0.294
SA origin	16.13	4.03	18.99	5.34	-2.069	0.039*

Termination of the Facial Artery

Of the 60 hemifaces, the type of origin and termination of the facial artery according to the classification of Bayram et al. is of Type I (99.5%), where it terminated as angular artery [28].

## Discussion

Multiple studies in the literature have discussed the site of origin, course, branching patterns, and their variations, as well as the distance between the origin of the branch from the main trunk to the reliable landmark on the face [2,6,7,16,21]. In 2015, Ahmed et al. described how the facial artery courses from origin to termination. They concluded that there is a difference in the branching pattern of facial arteries [4]. In 2022, Ramya dissected 50 cadavers to study the branches of the facial artery, and she found that in 82% of the cases, the facial artery terminates as an angular artery, which discusses the genesis and the termination of the facial artery branches on the face [9]. 

There are only few studies in the literature that have attempted to determine the diameter, course, and reliable location of each branch of the facial artery in the face [29,30]. However, there was none in the Indian population. So, the authors would like to bring attention to the fact that this study was conducted on cadavers of Indian origin by performing an extensive review of the literature, and comparisons were carried out in such a way that we were able to identify significant differences in length and origin of the facial branch in this study cohort as compared to the Indigenous population of other countries. Hence, it is important to acknowledge the difference in arterial morphology/topography based on ethnicity, and this is to be taken into account during the clinical application of the same. In the field of maxillofacial surgery, there are many reconstructive flaps that are based on the facial artery branches which include nasolabial flap, submental flap, Abbe-Estlander flap, and Karapandzic flap, and knowledge of the exact anatomy of the same is required to preserve their blood supply. As far as we can verify, this is the first study that has undertaken the determination of the linear and circumferential measurements of submental, superior labial, inferior labial, angular, and lateral nasal branches. 

In this study, we found that among these arteries, the inferior labial artery was the longest in the face with a measurement of 31.5 ± 7.86 mm, and this corresponds to the study conducted by Edizer et al. in the year 2003, which showed that inferior labial artery’s length was found to be 52.3 mm with a range of 16-98 mm. There is a significant difference from the study conducted by Edizer et al. which was done on Turkish cadavers. Similarly, there was a moderate difference seen in origin, with a measurement of 31.26 ± 6.4 mm in this study and 23.9 mm seen in the study by Edizer et al., and a mild difference in diameter, where 1.3 mm was noted in the present study and 1.2 mm was seen in the study by Edizer et al. [31].

The superior labial artery was found to be the most tortuous artery with a maximum diameter of about 1.68 ± 0.42, and when compared with the study accomplished by Magden et al. in the year 2004, the diameter was found to be 1.3 mm, showing a mild difference; there was a moderate difference in origin, with a measurement of 21.03 ± 9.8 mm in this study while the study by Magden et al. shows an origin of 12.1. There is a significant difference in the length (27.33 ± 9.17) found in the present study compared to that (45.4 mm, 29-85 mm) observed in the study by Magden et al. [23]. From the commissure of the lip, the superior labial artery lies closest with a distance of about 14.14 ± 5.05 mm. From the alar base, the lateral nasal artery runs closest with a distance of 1.15 ± 1.25.

In the neck, the submental artery showed a remarkable dissimilarity based on the genesis between the left and right sides with a p-value of 0.039 in this study. In the current study, the branching method of the facial artery was graded based on the study done by Bayram et al. in the year 2010: Type I, the facial artery terminating as the angular artery (76%); Type II, the facial artery terminating as the superior labial artery (12%); and Type III, the facial artery terminating as the inferior labial artery (12%). In comparison with the categories, this study shows the classification is of Type I (99.5%), where it terminated as the angular artery [28].

It is important to acknowledge that this is the first study that has undertaken determination of the linear and circumferential measurements of the submental, superior labial, inferior labial, angular, and lateral nasal branches of the facial artery. However, this study has certain limitations, including its single-center cadaveric design, small sample size, and being confined to a specific ethnic/racial group.

## Conclusions

This is one, if not the first, study of the facial artery branching patterns to be conducted on Indian-origin cadavers, and we believe it will help surgeons hone their reconstructive anatomy for the Indian population. Nuanced knowledge of its course, distribution, and detailed anatomy is necessary as it is the predominant vessel of the face. The study was carried out in such a way as to describe the length, location of origin, and branching pattern of each branch. These findings will enable surgeons to carry out meticulous surgery without damaging the facial artery branches. Furthermore, these findings will also help design new reconstructive local pedicled flaps, beyond that which is available in the literature at present. 
